# CHEK1 Expression Correlates with Tumor Progression in Lung Adenocarcinoma but Not in Squamous Cell Carcinoma

**DOI:** 10.3390/medicina62020335

**Published:** 2026-02-06

**Authors:** Nahyeon Kim, Hyunbin Cha, Jun-Chae Lee, Jae-Ho Lee, Tae-Young Kim

**Affiliations:** 1 Department of Anatomy, School of Medicine, Keimyung University, Daegu 42601, Republic of Korea; knh1621@naver.com (N.K.); hyunbincha@naver.com (H.C.); 2 Medical Course, School of Medicine, Keimyung University, Daegu 42601, Republic of Korea; chai1703@naver.com

**Keywords:** CHEK1, TCGA, lung adenocarcinoma, lung squamous cell carcinoma

## Abstract

*Background and Objectives:* Non-small cell lung cancer (NSCLC) is histologically divided into adenocarcinoma (AD) and squamous cell carcinoma (SCC). While Checkpoint kinase 1 (CHEK1) regulates the DNA damage response, its subtype-specific clinical impact in NSCLC remains unclear. We investigated the association of CHEK1 expression with clinicopathologic features and prognosis in AD and SCC. *Materials and Methods*: Transcriptomic and clinical data from 980 patients (492 AD, 488 SCC) were analyzed using The Cancer Genome Atlas (TCGA). Patients were stratified by median CHEK1 mRNA expression. Relationships between expression and clinicopathologic variables were evaluated via Chi-square tests, and overall survival (OS) was assessed using Kaplan–Meier analysis. *Results*: In AD, high CHEK1 expression significantly correlated with advanced T stage (*p* < 0.001), lymph node metastasis (*p* = 0.025), younger age (*p* = 0.017), and shorter OS (*p* = 0.025). Conversely, CHEK1 expression in SCC did not reach statistical significance for survival outcomes, although a borderline trend was observed (*p* = 0.067). *Conclusions*: CHEK1 is a subtype-specific prognostic biomarker for AD but not for SCC. These findings suggest that CHEK1 overexpression reflects tumor aggressiveness in AD, highlighting its potential as a therapeutic target for this specific population.

## 1. Introduction

Non-small cell lung cancer (NSCLC) accounts for approximately 85% of all lung cancer cases and remains the leading cause of cancer-related mortality worldwide [1,2]. NSCLC is histologically heterogeneous, primarily classified into lung adenocarcinoma (AD) and lung squamous cell carcinoma (SCC), which exhibit distinct cellular origins and clinical behaviors [3]. Although advances in targeted therapies have improved outcomes for specific subsets of patients, the prognosis for advanced NSCLC remains poor [4,5]. Comprehensive genomic profiling studies, including those by The Cancer Genome Atlas (TCGA), have delineated the unique molecular landscapes of these subtypes; for instance, EGFR mutations are predominant in AD, whereas SCC is characterized by TP53 mutations and FGFR1 amplification [6,7]. Consequently, identifying prognostic biomarkers that specifically correlate with clinicopathological features in each histological subtype is essential for developing precise therapeutic strategies based on the updated TNM classification [8]. Furthermore, lung AD and SCC arise from different cell types—distal alveolar type II cells and proximal airway basal cells, respectively—leading to fundamentally different metabolic demands and DNA repair dependencies [9,10]. Understanding these subtype-specific vulnerabilities is crucial for the clinical application of cell cycle inhibitors [4,11].

CHEK1 (Checkpoint kinase 1) is a conserved serine/threonine kinase that functions as a central effector of the DNA damage response (DDR) pathway, coordinating cell cycle arrest and DNA repair to maintain genomic integrity [9,12]. The DDR signaling network, including the ATR-CHEK1 axis, plays a pivotal role in monitoring replication stress, which is a hallmark of cancer cells driven by oncogenic proliferation [13,14,15]. Genomic instability and the deregulation of these checkpoint pathways are common features in tumorigenesis, allowing cancer cells to survive despite high levels of DNA damage [16,17].

Overexpression of cell cycle kinases like CHEK1 has been reported in a broad spectrum of malignancies. In addition to breast and colorectal cancers, high CHEK1 levels have been associated with poor prognosis and aggressive phenotypes in ovarian cancer [18] and pancreatic cancer [19], highlighting its potential as a pan-cancer therapeutic target [11,20]. In the context of NSCLC, high CHEK1 levels have been linked to aggressive tumor phenotypes and poor clinical outcomes [21,22]. Furthermore, preclinical evidence suggests that pharmacological inhibition of CHEK1 using agents such as AZD7762 or prexasertib (LY2606368) can sensitize lung cancer cells to DNA-damaging agents, highlighting its potential clinical utility [23,24,25,26]. From a mechanistic perspective, AD is frequently driven by oncogenic alterations such as EGFR or KRAS mutations, which are known to induce high levels of replication stress and thereby increase reliance on the ATR–CHEK1 axis for tumor cell survival. In contrast, SCC is characterized by near-universal TP53 mutations and complex genomic alterations, which may enable alternative checkpoint mechanisms and reduce dependence on CHEK1 signaling.

Despite these accumulating findings, the specific clinicopathological significance of CHEK1 expression may vary depending on the histological subtype of lung cancer. Most previous studies have evaluated NSCLC as a single entity or focused on general prognostic trends without distinguishing between subtypes. A detailed comparative analysis of how CHEK1 expression correlates with standard prognostic parameters—such as tumor stage and lymph node metastasis—specifically within AD versus SCC data remains to be fully elucidated. While the role of CHEK1 in general genomic stability is well-established [13,17], its specific contribution to the oncogenic signaling pathways that distinguish AD from SCC remains under-explored [27]. Additionally, established biomarkers such as EGFR and TERT play central roles in guiding clinical decision-making in lung cancer, serving as indicators of therapeutic response and tumor aggressiveness. In parallel, CHEK1 is emerging as a biomarker reflecting replication stress and DNA damage response dependency, suggesting its potential utility in identifying patients who may benefit from checkpoint-targeted therapies. In this study, we utilized large-scale transcriptomic data from the TCGA database to investigate the differential clinical relevance of CHEK1 expression in these two major histological subtypes of lung cancer.

## 2. Materials and Methods

### 2.1. TCGA Data Analysis

Primary data from The Cancer Genome Atlas (TCGA) data portal (http://cancergenome.nih.gov/ (accessed on 14 October 2025)) were downloaded. The data set consisted of a total of 980 NSCLC patients, including 492 with AD and 488 with SCC. To analyze the clinical significance of CHEK1, we extracted mRNA expression data along with clinical parameters, including age, sex, pathologic TNM stage, EGFR mutation status, and smoking status. This study met the publication guidelines for using TCGA data sets (https://portal.gdc.cancer.gov/ (accessed on 14 October 2025)).

### 2.2. Statistical Analysis

The Statistical Package for the Social Sciences (SPSS), version 26.0 for Windows (IBM, Armonk, NY, USA), was used for all statistical analyses. Patients were divided into high- and low-expression groups based on the median value of CHEK1 mRNA expression. The Chi-square test was used to analyze the relationship between CHEK1 expression and clinicopathological variables (age, sex, M stage, N stage, T stage, EGFR mutation, and smoking status). Survival analysis was performed using the Kaplan–Meier method, and the log-rank test was used to compare the overall survival differences between the two groups. A two-tailed *p* value < 0.05 was considered to signify statistical significance.

## 3. Result

### 3.1. Association of CHEK1 Expression with Clinicopathological Features in NSCLC

To elucidate the clinical relevance of CHEK1 in NSCLC, patients were stratified into high and low expression groups based on the median CHEK1 mRNA levels. The associations between CHEK1 expression and clinicopathological characteristics were evaluated separately for AD and lung SCC.

In the AD data, high CHEK1 expression was significantly correlated with aggressive clinicopathological features (Table 1). Specifically, we observed a progressive increase in the proportion of high CHEK1 expressions with advancing T stage between T stage and CHEK1 expressions; as the tumor size and local invasion increased from T1 to T3, the proportion of the high-expression group rose from 37.3% to 57.8% (*p* < 0.001). Statistical analysis revealed a significant association with tumor stage; the frequency of high CHEK1 expression increased progressively with advancing T stage (*p* < 0.001) and was significantly higher in patients with lymph node metastasis (≥ N1) compared to those without nodal involvement (57.7% vs. 46.9%; *p* = 0.025). Additionally, CHEK1 overexpression was significantly associated with younger age (≤ 65 years; *p* = 0.017) and male sex (*p* = 0.015). No statistically significant correlations were observed regarding M stage (*p* = 0.096), EGFR mutation status (*p* = 0.180), or smoking status (*p* = 0.629).

Conversely, in the SCC data, CHEK1 expression profiles displayed distinct clinical patterns (Table 2). Unlike AD, there were no significant correlations between CHEK1 expression and any of the examined clinicopathological parameters, including age (*p* = 0.642), sex (*p* = 0.836), pathologic TNM stage (*p* > 0.05 for all), EGFR mutation status (*p* = 0.435), or smoking status (*p* = 0.900).

### 3.2. Prognostic Significance of CHEK1 Expression in AD and SCC

**Figure 1:**
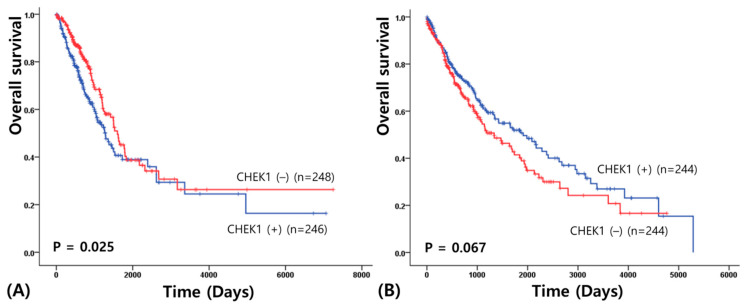
Overall survival analysis in lung adenocarcinoma (**A**) and squamous cell carcinoma (**B**) according to CHEK1 expression.

To determine the prognostic value of CHEK1, overall survival (OS) was assessed using Kaplan–Meier analysis and the log-rank test. In patients with AD, high CHEK1 expression was significantly associated with poor clinical outcomes. The high expression group exhibited a significantly shorter overall survival duration compared to the low expression group (2427.34 ± 318.60 vs. 2872.56 ± 345.45 days; χ^2^ = 5.01; *p* = 0.025) (Figure 1A). However, this prognostic impact was not observed in the SCC data. Although the survival curves showed a separation, the difference in overall survival between the high and low CHEK1 expression groups did not reach statistical significance (2307.62 ± 174.61 vs. 1880.64 ± 160.18 days; χ^2^ = 3.35; *p* = 0.067) (Figure 1B). Collectively, these findings suggest that CHEK1 overexpression serves as a subtype-specific prognostic biomarker for lung adenocarcinoma, reflecting its association with advanced tumor stages and aggressive biological behavior in this specific histological subtype.

## 4. Discussion

In this study, we investigated the clinicopathological and prognostic significance of CHEK1 expression in NSCLC using large-scale TCGA transcriptomic data. Our analysis provides a comprehensive comparison that emphasizes the histological divergence of CHEK1’s role, addressing the editor’s call for a more detailed examination of subtype-specific insights [26,28]. Our findings demonstrate that CHEK1 overexpression is significantly associated with aggressive tumor features and poor overall survival specifically in AD, but not in SCC. These results are consistent with recent bioinformatics analyses identifying CHEK1 as a hub gene related to poor prognosis in adenocarcinoma [25], suggesting that CHEK1 plays a distinct, subtype-specific biological role in the progression of lung adenocarcinoma.

CHEK1 is a critical regulator of the DNA damage response and cell cycle progression, ensuring genomic integrity by coordinating cell cycle arrest and DNA repair [9,10]. Consistent with its biological function, high CHEK1 expression correlates with high proliferation rates and therapy resistance in various cancers [15,18]. In our AD data, high CHEK1 expression was strongly correlated with advanced T stage and lymph node metastasis. This implies that CHEK1 may facilitate tumor growth and locoregional spread, potentially by enabling tumor cells to tolerate high levels of replication stress during rapid proliferation and invasion [12,21].

In our study, high CHEK1 expression in AD was significantly associated with advanced T stage, lymph node metastasis, and poor prognosis [29]. The observed dependency on CHEK1 in AD may be explained by the concept of synthetic lethality induced by oncogene-driven replication stress [14,20]. Although the survival difference in SCC did not reach statistical significance (*p* = 0.067), the observed trend suggests that CHEK1 may exert a modest effect in a subset of SCC patients. This borderline significance could be attributable to biological heterogeneity or limited statistical power, warranting further investigation in larger cohorts. While CHEK1 was a strong prognosticator in AD, it failed to reach statistical significance in SCC. This heterogeneity aligns with the distinct molecular landscapes of these tumors [26,27]. AD is often driven by oncogenic mutations in EGFR or KRAS, which induce substantial replication stress, thereby creating a dependency on CHEK1 for survival [6,23]. These oncogenes force cells into rapid S-phase entry, creating a heavy reliance on the ATR-CHEK1 axis to prevent the collapse of stalled replication forks [16,17]. In contrast, SCC is characterized by complex genomic rearrangements and near-universal TP53 mutations [7,29]. Large-scale prospective studies are needed to validate CHEK1 as an independent prognostic factor using multivariate survival analysis models [28,30,31]. It is plausible that SCC tumors utilize alternative survival mechanisms or possess redundant checkpoint pathways that render CHEK1 expression less rate-limiting for tumor progression compared to AD [32].

The association between high CHEK1 expression and younger age in AD is also noteworthy. Younger lung cancer patients often present with more aggressive disease and distinct genomic profiles, such as a higher prevalence of targetable driver mutations, compared to older patients [4,33]. The high CHEK1 levels in this demographic could reflect a more biologically active and proliferative tumor phenotype. Given that CHEK1 inhibitors, such as prexasertib, have shown promise in clinical trials [23,24] and can sensitize cancer cells to chemotherapy [16], our results suggest that younger AD patients with high CHEK1 expression might be the most suitable candidates for precision therapies involving CHEK1 inhibition.

There are some limitations in this study. First, the analysis is based solely on transcriptomic data derived from TCGA. While mRNA expression levels provide important insights, they do not always directly correlate with protein abundance or kinase activity. Validation at the protein level using immunohistochemistry (IHC) in independent patient data is necessary. Second, although we identified a statistical association between CHEK1 and poor prognosis, this study is retrospective and did not employ multivariate survival analysis models to adjust for confounders such as treatment history. Consequently, CHEK1 should be interpreted as a potential prognostic indicator rather than a confirmed independent prognostic factor at this stage, and large-scale prospective studies are needed to validate this [31]. Furthermore, in vitro experiments are required to explore the specific signaling pathways connecting CHEK1 to metastasis and invasion in lung adenocarcinoma [25]. Finally, the mechanistic basis for the differential role of CHEK1 in AD versus SCC was not experimentally explored. Future in vitro and in vivo experiments are required to elucidate the specific signaling pathways connecting CHEK1 to metastasis and invasion in lung adenocarcinoma.

## 5. Conclusions

In conclusion, our study demonstrates that CHEK1 expression serves as a distinct prognostic biomarker specifically for LUAD, but not for SCC. We identified that high CHEK1 expression in AD is significantly correlated with younger age, advanced tumor stages, and lymph node metastasis. Furthermore, high CHEK1 levels were associated with shorter overall survival in patients with AD, highlighting its potential utility in stratifying high-risk patients. These findings suggest that CHEK1 may be a promising therapeutic target for adenocarcinoma, particularly in younger patients or those with aggressive disease features. Clinically, CHEK1 expression may serve as a useful biomarker to identify high-risk AD patients who could benefit from intensified surveillance or emerging CHEK1-targeted therapies. Future studies should focus on validating these findings at the protein level and exploring the efficacy of CHEK1 inhibitors in biomarker-defined AD data.

## Figures and Tables

**Table 1:** Clinical characteristics of CHEK1 expression in lung adenocarcinoma.

	CHEK1 Expression		*p* Value
	High (%, N)	Low (%, N)	
Age			0.017
≤65 years	55.5 (131)	44.5 (105)	
≥65 years	44.7 (114)	55.3 (141)	
Sex			0.015
Male	56.0 (126)	44.0 (99)	
Female	44.9 (120)	55.1 (147)	
M stage			0.096
M0	49.1 (159)	50.9 (165)	
M1	66.7 (16)	33.3 (8)	
N stage			0.025
N0	46.9 (149)	53.1 (169)	
≥N1	57.7 (94)	42.3 (69)	
T stage			<0.001
T1	37.3 (62)	62.7 (104)	
T2	56.9 (148)	43.1 (112)	
T3	57.8 (26)	42.2 (19)	
T4	44.4 (8)	55.6 (10)	
EGFR mutation			0.180
(+)	34.5 (10)	65.5 (19)	
(−)	47.8 (88)	52.2 (96)	
Smoking status			0.629
Yes	69.1 (170)	67.1(165)	
No	30.9 (76)	32.9 (81)	

**Table 2:** Clinical characteristics of CHEK1 expression in lung squamous cell carcinoma.

	CHEK1 Expression		*p* Value
	High (%, N)	Low (%, N)	
Age			0.642
≤65 years	48.7 (92)	51.3 (97)	
≥65 years	50.8 (152)	49.2 (147)	
Sex			0.836
Male	50.3 (182)	49.7 (180)	
Female	49.2 (62)	50.8 (64)	
M stage			0.59
M0	53.1 (213)	46.9 (188)	
M1	42.9 (3)	57.1 (4)	
N stage			0.973
N0	49.8 (155)	50.2 (156)	
≥N1	50.9 (87)	49.1 (84)	
T stage			0.949
T1	48.6 (53)	51.4 (56)	
T2	50.0 (143)	50.0 (143)	
T3	52.9 (37)	47.1 (33)	
T4	47.8 (11)	52.2 (12)	
EGFR mutation			0.435
(+)	42.9 (9)	57.1 (12)	
(−)	48.2 (131)	51.8 (141)	
Smoking status			0.900
Yes	42.2 (206)	42.4 (207)	
No	7.8 (38)	7.6 (37)	

## Data Availability

The data presented in this study are available on request from the corresponding author.
